# Interactions’ quality among healthcare professionals and associated factors

**DOI:** 10.1192/j.eurpsy.2026.11719

**Published:** 2026-07-31

**Authors:** A. Sdiri, I. Betbout, M. Harran, H. Zaalouni, N. Faouel, M. Ben Mbarek, A. Ben Haouala, B. Amamou, A. Mhalla

**Affiliations:** 1 Psychiatry Research Laboratory LR05ES10 (Vulnerability To Psychoses), Faculty Of Medicine Of Monastir, University Of Monastir; 2 Psychiatry, Fattouma Bourguiba university hospital, Monastir; 3 UPSAT, Sousse, Tunisia

## Abstract

**Introduction:**

Healthcare institutions need to improve care integration and coordination, especially in response to economic and technological changes. Good teamwork and clear communication, supported by a strong ethical environment, help improve the quality and continuity of care. Work organization and a shared sense of purpose directly influence team performance.

**Objectives:**

The aim of this study is to evaluate the quality of interactions among healthcare professionals’ and to highlight factors influencing the continuity and effectiveness of patient care.

**Methods:**

This descriptive cross-sectional qualitative study was conducted from January to March 2025 among healthcare professionals working in the emergency and intensive care units of three university hospitals in Tunisia, using a self-administered questionnaire.

**Results:**

The majority of respondents were female (78.4%) with a sex ratio of 0.28, with over half being under the age of 30 (56.8%). Most held the position of nurse (75.2%), among whom 70.2% were senior nurses. Nearly two-thirds had less than five years of professional experience (64.0%) and mainly worked morning shifts (56.8%). A large majority had obtained their degrees after 2006 (86.4%) and worked in intensive care units (57.6%). Most respondents reported working six hours per day (91.2%), and over half were single (55.2%). Regarding work organization, 73.6% considered it acceptable, and 70.4% rated teamwork collaboration as satisfactory. Interpersonal communication in the workplace was rated as average (46.4%) or good (39.2%) by most respondents. The most frequently reported barriers were stress and fatigue (74.4%). Additionally, 67.2% believed that staffing levels were moderately insufficient. Verbal communication was the most commonly used channel (85.6%), while communication interruptions were perceived as frequent (45.6%) or occasional (24.8%).

**Conclusions:**

Interpersonal communication is influenced by work organization and management. Workload pressure, stress, and understaffing can lead to misunderstandings, particularly in the continuity of care. These findings highlight the need for increased attention to communication dynamics and staffing levels within healthcare institutions.

**Disclosure of Interest:**

None Declared

